# Validation of the Korean Version of the Health Care Climate Questionnaire among Cancer Survivors

**DOI:** 10.3390/healthcare12030323

**Published:** 2024-01-26

**Authors:** Hyun-E Yeom, Jungmin Lee, Young-Joo Kim

**Affiliations:** 1 Department of Nursing, Chungnam National University, Munhwaro 266, Junggu, Daejeon 35015, Republic of Korea; yeom@cnu.ac.kr; 2 Korea Research Institute for Vocational Education & Training, Social Policy Building, Sejong National Research Complex, 370 Sicheong-daero, Sejong-si 30147, Republic of Korea; 3 Department of Economics, Hongik University, Seoul 04066, Republic of Korea

**Keywords:** chronic illnesses, health care climate questionnaire, supportive environment

## Abstract

Healthcare professionals should support autonomy in their patients in order for them to maintain the motivation to cope actively with their conditions. The Health Care Climate Questionnaire (HCCQ) is useful for assessing patients’ perceptions of the autonomy support provided to them. We aimed to validate the psychometric properties of the Korean version of the HCCQ (HCCQ-K) among Korean cancer survivors. This study evaluated the factor structure, concurrent validity, and internal consistency. Data from 367 cancer survivors were analyzed using confirmatory factor analysis (CFA), Pearson’s correlations, and Cronbach’s α values. The CFA validated that the single-factor structure of the HCCQ-K had an excellent fit that was consistent with that of the original English version. Concurrent validity was confirmed by moderate correlations between the HCCQ-K and both psychological well-being and self-management. Reliability was verified by satisfactory internal consistency, with a Cronbach’s α value of 0.91 and strong item-total and inter-item correlations. The HCCQ-K is therefore a valid and reliable tool for assessing autonomy support provided by healthcare professionals to Korean cancer survivors. The HCCQ-K may help healthcare professionals understand their patients’ needs for autonomy support and develop strategies to motivate active coping behaviors.

## 1. Introduction

Progress in medical technology has played a pivotal role in increasing cancer survival rates, resulting in cancer now often being characterized as a chronic health concern [1]. Cancer survivors encounter persistent challenges to engaging in proactive self-care—including adherence to medical regimens, dietary regulations, and physical activity—all amid uncertainties surrounding potential cancer recurrence and exacerbation [1,2].

Autonomy support from healthcare professionals is a central issue related to behavioral and psychological well-being among individuals with chronic health concerns [3,4]. Autonomy entails recognizing a sense of choice and volition in actions, reflecting autonomous decision-making and voluntary participation with independence and personal authority [3,4,5,6]. Grounded in the Self-Determination Theory (SDT), autonomy, which has been deemed a basic psychological need, is pivotal for fostering motivation, behavioral changes, and accomplishments [5,6,7]. In healthcare settings, patient autonomy signifies a sense of self-determined behavior, distinct from external pressures or control exerted by healthcare professionals [7,8,9,10,11].

Healthcare research highlights that a sense of autonomy in healthcare contexts is achieved when healthcare professionals acknowledge patients’ perspectives and provide relevant information, respect, and understanding [3,4,5,6,7]. In agreement with the theoretical propositions of the SDT, empirical studies have shown that patients’ perspectives on the autonomy support from healthcare professionals affect the ownership, responsibility, and internalization of their own behavior, leading to more active engagement in self-care and better health outcomes [5,6,7,8,9,10]. Therefore, it is essential to understand patients’ perceptions of autonomy support from healthcare professionals for facilitating intrinsic motivation towards self-determined behavioral changes and engagement. 

The Health Care Climate Questionnaire (HCCQ) is a valid tool for gauging individuals’ perceptions of autonomy support received from their healthcare professionals [5,6,12,13,14,15]. This perception involves recognizing autonomous responsibility and self-directed behavior when managing health concerns. The HCCQ questions specifically inquire about individuals’ experiences of perceiving supportive attitudes when determining and considering their preferences for coping with health concerns [5,6]. The original HCCQ and its translations into French [13], German [14], and Persian [15] have been used to explore autonomy support perceptions among patients with diverse health issues such as cancer, type 2 diabetes, cardiovascular disease, and chronic kidney disease [12,13,14,15]. Several studies have investigated the concept of autonomy support within clinical healthcare contexts for the Korean population with acute or chronic health problems [8,16,17,18,19], thus revealing associations with both empirically and conceptually relevant variables, including self-efficacy, motivation, self-care, and adherence to medical regimens. Also, one study [20] validated the modified Korean version of HCCQ, customized to harmonize with the specific attributes of the psychological counseling environment. However, the psychometric properties of the HCCQ for the Korean population remain unexplored in clinical healthcare contexts. 

Autonomy support from healthcare professionals is highly significant, especially for cancer survivors, as it serves as a consistent source of internal empowerment amid the uncertainty and anxiety surrounding cancer recurrence and exacerbation. With the increasing number of cancer survivors, it is imperative to examine the psychometric properties of the HCCQ-K thoroughly. This study has the potential to enhance our understanding of the autonomous responsibilities experienced by cancer survivors in their interactions with healthcare professionals, thereby facilitating active self-care and fostering improved health outcomes. Therefore, we aimed to investigate the psychometric properties of the HCCQ-K and validate its construct validity and internal consistency within the Korean population, with a specific focus on cancer survivors. 

## 2. Materials and Methods

### 2.1. Design

This study applied a methodological approach to identify the conceptual structures underlying the HCCQ-K by using merged data from two cross-sectional studies involving cancer survivors.

### 2.2. Sample and Process

The study included 367 cancer survivors drawn from two independent studies (Studies 1 and 2). Study 1 aimed to explore the associations between psycho-cognitive and behavioral factors and quality of life in 220 breast cancer survivors. Focusing on three dimensions of basic psychological needs, including autonomy, a cross-sectional study was conducted to investigate how the psychological needs are associated with social support, self-care, and the quality of life of breast cancer survivors. Study 2 was designed to investigate the psychosocial factors related to self-care among 147 blood cancer survivors. Specifically, Study 2 investigated the influence of social support, including family and health professionals, on self-care in blood cancer survivors. 

Data for both studies were collected through self-administered surveys at a cancer center affiliated with a university hospital in the central region of the Republic of Korea. The inclusion criteria for both studies were as follows: participants were aged at least 19 years, aware of their cancer diagnoses, had completed and/or were in follow-up stages after active cancer treatment (e.g., chemotherapy, radiation therapy, surgery), had no cognitive impairments, and voluntarily agreed to participate. 

Based on these criteria, the total number of participants recruited was 367, with 220 breast cancer survivors from Study 1 and 147 blood cancer survivors from Study 2. A sufficient sample size for examining factor structures via confirmatory factor analysis (CFA) is recommended to maintain a ratio of cases to free parameters within the range of 10:1 to 20:1 [21,22]. Given that the HCCQ-K consists of six items, the requisite sample size should fall within the range of 60 to 120, in accordance with the specified criteria. Therefore, the sample size supported construct validity with sufficient statistical power.

### 2.3. Translation into the Korean Version of HCCQ

The original HCCQ underwent a comprehensive cross-cultural adaptation process for self-administered use within the context of Korean sociocultural norms. The adaptation process involved five steps: translation, synthesis, back-translation, content validity review, and pre-testing [23].

In the initial translation step, two bilingual experts in Korean and English created the initial Korean version of the HCCQ to ensure linguistic accuracy and cultural relevance. Individuals facing chronic health issues, including cancer, can access a range of medical services, from private clinics to tertiary general hospitals within the Korean healthcare system, engaging with a diverse array of healthcare professionals. In particular, cancer survivors maintain interactions with various healthcare professionals during extended therapeutic and follow-up periods. Consequently, given the characteristics of the Korean healthcare context, the term “physician” was translated to “healthcare professional.” In the content synthesis step, a team of two specialists in nursing and psychoeducation and two clinical healthcare professionals assessed the initial translation for comprehensibility and alignment with Korean cultural norms. Another bilingual professional conducted a meticulous back-translation to identify any discrepancies and ensure translation harmony while preserving the questionnaire’s content integrity. A panel of experts in nursing research and healthcare professionals assessed content validity, focusing on cultural and linguistic adaptation, further enhancing the questionnaire’s suitability in the Korean context. Finally, a pre-testing phase involving 20 breast cancer survivors evaluated the questionnaire’s clarity and identified potential participants’ difficulties. Breast cancer survivors were chosen since breast cancer ranks as the predominant cancer among Korean women, boasting a notably high survival rate. The intentional diversity observed within the sample, encompassing individuals undergoing various treatments, such as surgery, radiation, and chemotherapy, is strategically designed to encapsulate the representative spectrum of cancer survivors. Following the successful completion of these rigorous steps, the HCCQ-K was refined and finalized, resulting in a six-item questionnaire tailored to the unique sociocultural context of Korea (see Supplementary). 

### 2.4. Measures

#### 2.4.1. Psychological Well-Being

The psychological well-being of breast cancer survivors was measured using seven items related to purpose in life as one of the six dimensions of the Psychological Well-Being scales [24,25]. The concept of purpose in life refers to an individual’s sense of directionality and goals. It also encompasses the feeling that there is meaning in one’s present and past lives and that the individual has objectives in their life. Participants rated their agreement with each item on a 6-point Likert scale ranging from 1 (strongly disagree) to 6 (strongly agree). A higher score indicated a greater positive attitude toward one’s life. The internal consistency of Study 1 was assessed to have a Cronbach’s α of 0.809. 

The Uncertainty in Illness Scale was measured for the psychological attributes of blood cancer survivors using Mishel’s Uncertainty in Illness Scale [26], which has previously been validated in Korean cancer patients [27]. The scale consists of 23 items rated on a 5-point Likert scale ranging from 1 (strongly disagree) to 5 (strongly agree). The sum of the items is scored, with a higher score indicating greater ambiguity and complexity regarding health issues. The reliability of a prior study about Korean cancer survivors [27] was reported at Cronbach’s α levels of 0.85. The internal consistency of Study 2 was assessed as having a Cronbach’s α of 0.837.

#### 2.4.2. Health-Related Self-Management 

Engagement in self-management of breast cancer was assessed using the Health Promoting Lifestyle Profile (HPLP) II questionnaire [28]. As a valid measure for evaluating overall and division-specific engagement in healthy behaviors across diverse countries, it has been validated in diverse Korean populations [29], including cancer survivors [30]. The HPLP II consists of 52 items divided into six subdomains: physical activity (8 items), health responsibility (9 items), nutrition (9 items), spiritual growth (9 items), interpersonal relations (9 items), and stress management (8 items). Participants rate the frequency of each item on a 4-point Likert scale ranging from 1 (never), to 2 (sometimes), 3 (often), and finally 4 (routinely). The total score is calculated for all items, resulting in a score range of 52–208 points, with higher scores indicating more proactive engagement in health behaviors. The internal consistency in previous studies ranged from 0.947–0.951 regarding Cronbach’s α. The reliability of the current study was Cronbach’s α = 0.957. 

Engagement in health-related self-management was evaluated using a self-care scale that was developed and revised for Korean blood cancer survivors [31]. The instrument consists of 19 items that reflect behaviors related to managing infection risk (11 items), bleeding risk (4 items), and nutrition management (4 items). Each item is measured using a 4-point Likert scale ranging from 1 (never) to 2 (sometimes), 3 (usually), and finally 4 (always) in terms of the frequency with which participants engage in self-care in daily living. The scores for self-care behaviors range from 19–76 points, with higher scores indicating more active self-care practices. The reliability in the original study [31] was reported as Cronbach’s α levels of 0.850 and 0.910, respectively. In this study, reliability was assessed as having a Cronbach’s α of 0.889. 

#### 2.4.3. Demographic and Cancer-Related Characteristics

Sociodemographic data, including age, educational level, income level, current employment status, and living arrangements, were collected. Regarding past medical history related to cancer, information on post-diagnosis periods, specific treatments received, and any concurrent comorbid conditions was also documented.

### 2.5. Ethical Consideration

The Institutional Review Boards approved the purposes and procedures for both original studies (approval numbers 202101-SB-009-01 for Study 1 and CNUH 2021-06-080-006 for Study 2). In both studies, participants were provided a thorough explanation of the study’s objectives, procedures, ethical considerations, and the rights guaranteed to them as study participants. Each one then indicated their voluntary participation in the study by signing an informed consent document.

### 2.6. Statistical Analysis 

Data analysis was performed using SPSS version 26.0 (IBM Corp., Armonk, NY, USA) and Mplus version 7.0 (Muthen & Muthen, Los Angeles, CA, USA). Preliminary and descriptive statistics were calculated to provide an overview of the demographic and clinical characteristics of the participants, as well as the primary variables in the study. The psychometric properties of the HCCQ-K were examined with respect to its construct validity—including structural validity, concurrent validity, and internal consistency reliability. 

Specifically, the construct validity of the single-factor structure of the HCCQ-K was tested using CFA with maximum likelihood estimation. The goodness of fit was evaluated using multiple indexes: χ^2^/ԁƒ (<3), Comparative Fit Index (CFI; >0.90), Tucker-Lewis Index (TLI; >0.90), root-mean-square error of approximation (RMSEA; <0.08), and standardized root-mean-square residual (SRMR; <0.08) [32,33], as well as the values of AVE (>0.5) and CR (≥0.6) [33]. 

Concurrent validity, a component of construct validity, was assessed through a two-part analysis. We explored the associations of the HCCQ-K with two dimensions—psychological well-being and self-management—guided by both theoretical foundations and empirical evidence. Regarding psychological well-being, Pearson’s correlation coefficients were employed to examine the connections between the HCCQ-K and sense of purpose in life, as well as the level of uncertainty. In the realm of self-management, Pearson’s correlation coefficients were utilized to assess the relationships of the HCCQ-K with health-promoting and self-care behaviors.

Internal consistency reliability was examined with Cronbach’s α coefficients for the HCCQ-K, as well as item-total and inter-item correlations. A Cronbach’s α value of ≥0.70 is indicative of an acceptable level of reliability, signifying that the items effectively measure the same underlying construct [33,34]. Acceptable ranges for item-total correlations (ranging from 0.30–0.80) and inter-item correlations (within 0.15–0.50) were taken into consideration [35,36].

## 3. Results

### 3.1. Participant Characteristics

Table 1 illustrates the sociodemographic and cancer-related characteristics of the participants. They had an average age of 54.87 years (standard deviation [SD] = 11.78). The majority were married and had an educational background beyond high school graduation. 

The study participants were diagnosed with two distinct types of cancer: malignant neoplasms of the breast (n = 220) and malignant neoplasms of lymphoid, hematopoietic, and related tissue (n = 147). Further details regarding the specific types within the latter group are presented in Table 1, revealing a predominant occurrence of leukemias (n = 123). The average duration since the initial cancer diagnosis was 25.41 months (SD = 22.16). In terms of therapeutic modalities, most of the breast cancer survivors (n = 188) underwent lumpectomy or mastectomy. Out of the participants with blood cancer, 127 survivors, constituting approximately half of the cohort, received exclusive chemotherapy, and 53 survivors underwent hematopoietic stem cell transplantation. 

### 3.2. Construct Validity 

#### 3.2.1. Structural Validity

The CFA confirmed the goodness-of-fit of the single-factor structure of the HCCQ-K (Table 2 and Table 3). All six items of the HCCQ-K were allocated to one dimension, which was the same as the original structure, with factor loadings ranging from 0.693–0.920. The model showed an excellent fit with the data: CFI = 0.995; TLI = 0.990; RMSEA = 0.006; 95% confidence interval as 0.019, 0.097; SRMR = 0.012; χ^2^/dƒ = 15.813; and *p* = 0.027. The average variance extracted (AVE = 0.693) and composite reliability (CR = 0.931) values were above the acceptable values. 

#### 3.2.2. Criterion-Related Concurrent Validity

Table 4 displays the relationship between the total score of the HCCQ-K and the two dimensions of psychological well-being and health-related self-management, which are theoretically and empirically relevant to patients’ autonomy support. The HCCQ-K was significantly related to psychological well-being, which assesses the level of having a purpose in life (r = 0.290, *p* = 0.000) in breast cancer survivors and uncertainty (r = −0.223, *p* = 0.007) in blood cancer survivors. Regarding self-management behaviors, the HCCQ-K was correlated with levels of health-promoting behaviors (r = 0.281, *p* = 0.000) in breast cancer survivors and self-care (r = 0.313, *p* = 0.000) in blood cancer survivors. 

### 3.3. Internal Consistency Reliability

The internal consistency for the HCCQ-K was sufficient, at a Cronbach’s α of 0.91, which exceeded the acceptable threshold. 

The item-total scale correlation coefficient for the six items (Table 5) was greater than 0.3, indicating good internal consistency. The item-to-item correlation coefficients ranged from 0.546–0.828, with an average item-to-item correlation of 0.702. The result was satisfactory for the criteria over an adequate range (0.300 ≤ r ≤ 0.900). 

## 4. Discussion

Understanding the experiences of cancer survivors concerning autonomy support is crucial for promoting proactive self-care and improving health outcomes. This study established the validity and reliability of the HCCQ-K for assessing cancer survivors’ perceptions of autonomy support provided by healthcare professionals in Korea. 

In alignment with the original HCCQ, our CFA demonstrated that the factor structure underlying the HCCQ-K was unidimensional. The six items constituting the HCCQ-K were equally valuable in evaluating the patients’ perceptions of autonomy support provided to them by their healthcare professionals, as demonstrated by the factor loading values that exceeded 0.6.

Our study further verified the construct validity of the HCCQ-K, establishing a close relationship with both theoretically and empirically relevant factors encompassing psychological well-being and self-management. Empirical studies grounded in SDT have shown that the fulfillment of an individual’s autonomy is linked to increased intrinsic motivation, well-being, and personal growth [7,8,9,10,11]. Conversely, individuals may encounter adverse outcomes such as diminished motivation and psychological distress when their autonomy needs are frustrated or unmet [7,8].

In accordance with the theoretical tenets of SDT [3,4,7], our findings underscore the importance of autonomous support from healthcare professionals. Cancer survivors who received greater autonomy support from healthcare professionals tended to have a stronger sense of purpose in life and less uncertainty. They were also more inclined to participate in health-promoting and self-care behaviors. Focusing on the autonomy of the basic psychological needs of SDT, this study clarified why autonomy support from healthcare professionals is crucial and what forms of support could be advantageous for patients’ self-care and quality of life. 

This study confirmed the reliability of the HCCQ-K as a measurement tool, demonstrating a high level of internal consistency and composite reliability across its six items that surpassed the acceptable criterion of 0.7 [33]. The HCCQ-K establishes a robust positive relationship between each individual item and its respective score, as well as with the overall HCCQ-K score. Moreover, all six items exhibited reciprocal correlations, with coefficient values ranging from 0.546–0.828.

The original HCCQ exists in two forms [5,6,12,13,14,15]: one comprises 15 items, and the other is the abbreviated counterpart composed of six items, which formed the basis for the development of the HCCQ-K. Our findings showed that this abbreviated version was adequate for evaluating cancer survivors’ perceptions of autonomy support received from healthcare professionals. Using the six-item HCCQ-K scale is both time-efficient and advantageous in reducing participants’ burden and facilitating a more precise assessment of genuine cognitive responses.

While the significance of autonomy support is universally acknowledged [3,4,7], variations in perspectives may arise due to cultural and sociocultural contexts and differences in healthcare systems [37]. The findings of this study affirm the importance of autonomy support from healthcare professionals, as measured by the HCCQ-K, for Korean cancer survivors. This endorsement holds value within the framework of cognitive-behavioral studies that seek to explore strategies for enhancing health outcomes in this population. 

Nevertheless, it is crucial to acknowledge the specific limitations inherent in the current study. The intricate nature of cancer, which is characterized as a chronic condition [1,2], introduces variability in perspectives on autonomy support among cancer survivors. This is influenced by factors such as cancer type, therapeutic procedures, survival rates, and disease stages. Additionally, viewing autonomy as a basic psychological need for individuals [3,4,6], its perception may be intricately shaped by the health-related sociodemographic contexts within cancer populations. Given the potential diversity in autonomy-related perspectives, the study’s findings, limited to blood and breast cancer, may not readily generalize to the entire population of cancer survivors in Korea. Consequently, the enhancement of HCCQ-K applicability necessitates further replicative studies.

Moreover, as underscored in prior research, the perceptions of autonomy support among patients play pivotal roles in regulating coping behaviors and influencing psychosocial and physiological health outcomes across various health-related challenges [7,8,9,16,17,18,19,20]. Consequently, further research is warranted, applying the HCCQ-K to explore SDT-based cognitive-behavioral mechanisms among individuals with diverse health conditions beyond cancer. This would contribute to confirming the psychometric properties of the HCCQ-K within the Korean population.

## 5. Conclusions

This study demonstrates that the HCCQ-K is a valid and reliable instrument for assessing cancer survivors’ perceptions of autonomy support provided by healthcare professionals in Korea. The use of the HCCQ-K is beneficial for understanding the needs of cancer survivors and offers insights into promoting self-directed care. Subsequent research, encompassing survivors of various cancer types beyond blood and breast cancer, is essential to enhancing the generalizability of evidence validating the HCCQ-K. Furthermore, an exploration into the theoretical propositions of SDT concerning the role of autonomy support, as assessed by the HCCQ-K, is justified to further augment its applicability.

## Figures and Tables

**Table 1:** Descriptive characteristics of the participants (N = 367).

Variables	Categories	M ± SD or n (%)
Age		54.87 ± 11.78
Gender	Male	82 (22.3)
	Female	285 (77.7)
Education	≤Middle school graduation	68 (18.5)
	High school graduation	133 (36.3)
	≥College	166 (45.2)
Marital status	Married	293 (79.8)
	Widowed or divorced	39 (10.6)
	Unmarried	35 (9.6)
Living status	Living with family or someone else	334 (91.1)
	Living alone	33 (8.9)
Currently employed	Yes	167 (45.5)
Monthly household income	<200	109 (29.7)
(10,000 KRW ^1^/month)	200–299	72 (19.6)
	300–399	58 (15.8)
	≥400	128 (34.9)
Types of cancer	Malignant neoplasms of the breast	220
	Leukemias	123
	Hodgkin or non-Hodgkin lymphoma	17
	Multiple myeloma	7
Therapeutic history	Surgery	188 (51.2)
	Chemotherapy	264 (71.9)
	Hormone therapy	82 (22.3)
	HSCT ^2^	53 (14.4)
	Others (e.g., radiation, immunotherapy)	40 (10.9)
Duration since cancer diagnosis (months)		25.41 ± 22.16

Note. ^1^ KRW: Korea won; ^2^ HSCT: hematopoietic stem cell transplantation; M: mean; SD: standard deviation.

**Table 2:** Factor structure of the HCCQ-K (N = 367).

Items	FL	EE	AVE	CR
1. I feel that my healthcare provider has provided me choices and options.	0.693	0.030		
2. I feel understood by my healthcare providers.	0.920	0.011		
3. My healthcare provider conveys confidence in my ability to make changes.	0.874	0.015	0.693	0.931
4. My healthcare provider encourages me to ask questions.	0.796	0.022		
5. My healthcare provider listens to how I would like to do things.	0.849	0.017		
6. My healthcare provider tries to understand how I see things before suggesting a new way to do things.	0.844	0.018		
Variance explained (%) Total = 60.8				

Note. FL: factor loading; EE: error estimate; AVE: average variance extracted; CR: composite reliability.

**Table 3:** The main results of the model fit by the confirmatory factor analysis (N = 367).

Metric	Estimate	Threshold	Interpretation
χ^2^ (*df*)	15.813 (7)	*p* = 0.0269	Pass
CFI ^1^	0.995	>0.900	Pass
TLI ^2^	0.990	>0.900	Pass
RMSEA ^3^ (95% CI)	0.059 (0.019 0.097)	<0.100	Pass
SRMR ^4^	0.012	<0.080	Pass

Note. ^1^ CFI: comparative fit index; ^2^ TLI: Tucker–Lewis index; ^3^ RMSEA: root mean square error of approximation; ^4^ SRMR: standardized root mean square residual.

**Table 4:** Relationships of the HCCQ-K with theoretically relevant concepts.

	Psychological Well-Being		Self-Management	
	Purpose in Life ^1^ *r* (*p*)	Uncertainty ^2^ *r* (*p*)	Health-Promoting Behavior ^1^ *r* (*p*)	Self-Care ^2^ *r* (*p*)
HCCQ-K	0.290 (0.000)	−0.223 (0.007)	0.281 (0.000)	0.313 (0.000)

Note. HCCQ-K: Health Care Climate Questionnaire-Korean; ^1^ Study 1 for 220 breast cancer survivors; ^2^ Study 2 for 147 blood cancer survivors.

**Table 5:** HCCQ-K item means, standard deviations, and correlations of the total score (N = 367).

Items	M (SD)	Corrected Item-Total *r* (*p*)	Item-Total *r* (*p*)	Cronbach’s Alpha if the Item Deleted
1. I feel that my healthcare provider has provided me choices and options	5.40 (1.73)	0.671	0.778 (0.000)	0.937
2. I feel understood by my healthcare providers	5.42 (1.57)	0.872	0.913 (0.000)	0.909
3. My healthcare provider conveys confidence in my ability to make changes	5.31 (1.54)	0.842	0.892 (0.000)	0.913
4. My healthcare provider encourages me to ask questions	5.79 (1.57)	0.793	0.858 (0.000)	0.920
5. My healthcare provider listens to how I would like to do things	5.70 (1.48)	0.837	0.887 (0.000)	0.914
6. My healthcare provider tries to understand how I see things before suggesting a new way to do things	5.25 (1.68)	0.800	0.867 (0.000)	0.919

Note. M: mean; SD: standard deviation.

## Data Availability

The data supporting this study’s findings are not publicly available since participants did not give written consent for their data to be shared publicly.

## Supplementary Materials

The following supporting information can be downloaded at: https://www.mdpi.com/article/10.3390/healthcare12030323/s1, Table S1. Six Items of the Original and Korean Versions of the Health Care Climate Questionnaire.
