# Supplementary material for: Validation of the Korean Version of the Health Care Climate Questionnaire among Cancer Survivors
Source: Healthcare (Basel). 2024 Jan 26;12(3):323. doi: 10.3390/healthcare12030323 (PMC10855044; doi:10.3390/healthcare12030323)
Supplement: Supplementary file 1 [file healthcare-12-00323-s001.zip › healthcare-2788362-supplementary.pdf]

Supplementary Table S1. Six Items of the Original and Korean Versions of the Health Care Climate Questionnaire.

English	Korean
1. I feel that my healthcare provider has provided me choices and options	의료진이 내게 선택할 기회를 준다고 느낀다.
2. I feel understood by my healthcare providers	의료진이 나를 이해하고 있다고 느낀다.
3. My healthcare provider conveys confidence in my ability to make changes	의료진은 내가 변화할 수 있는 힘이 있다고 믿어준다.
4. My healthcare provider encourages me to ask questions	의료진은 내가 궁금한 것을 물어볼 수 있게 해준다.
5. My healthcare provider listens to how I would like to do things	의료진은 내가 어떻게 하길 원하는지 듣는다.
6. My healthcare provider tries to understand how I see things before suggesting a new way to do things	의료진은 어떤 새로운 제안을 하기 전에 내가 어떻게 생각하는지 이해하려고 노력한다.
